# Supplementary material for: Carbon-anchoring synthesis of Pt1Ni1@Pt/C core-shell catalysts for stable oxygen reduction reaction
Source: Nat Commun. 2024 Nov 1;15:9458. doi: 10.1038/s41467-024-53808-y (PMC11530681; doi:10.1038/s41467-024-53808-y)
Supplement: Supplementary file 1 — Supplementary Information [file 41467_2024_53808_MOESM1_ESM.pdf]

Supplementary Information

Carbon-anchoring synthesis of Pt_1Ni_1@Pt/C core-shell catalysts for stable oxygen reduction reaction

Jialin Cui[1,†], Di Zhang[2,†], Zhongliang Liu[1,†], Congcong Li[1], Tingting Zhang[1], Shixin Yin[1], Yiting Song[1], Hao Li[2,*], Huihui Li[1,*], Chunzhong Li[1,3,*]

[1]*Key Laboratory for Ultrafine Materials of Ministry of Education, School of Chemical Engineering, East China University of Science and Technology, Shanghai 200237, China.*

[2]*Advanced Institute for Materials Research (WPI-AIMR), Tohoku University, Sendai 980–8577, Japan*

[3]*Shanghai Engineering Research Center of Hierarchical Nanomaterials, School of Materials Science and Engineering, East China University of Science and Technology, Shanghai 200237, China.*

*Correspondence and requests for materials should be addressed to Hao Li (li.hao.b8@tohoku.ac.jp), Huihui Li (huihuili@ecust.edu.cn), Chunzhong Li (czli@ecust.edu.cn).

†These authors contributed equally to this work.

Supplementary methods

Materials and chemicals. Chloroplatinic acid hexahydrate ($H_2PtCl_6 \cdot 6H_2O$, ≥37.5% Pt basis), nickel (II) nitrate hexahydrate ($Ni(NO_3)_2 \cdot 6H_2O$, ≥99.5%), ethylene glycol ($C_2H_6O_2$, ≥99%), and sodium hydroxide (NaOH, ≥99%) were purchased from Aladdin. 2-propanol (C_3H_8O, HPLC, 99.9%) was purchased from Honeywell. Carbon black (Vulcan XC-72) was purchased from Cabot. Ethanol (C_2H_6O, ≥99.7%), perchloric acid ($HClO_4$, 70~72 wt. %), and acetone (C_3H_6O, ≥99%) were purchased from General-Reagent. Perfluorinated resin solution containing Nafion™ 1100W (5 wt. % in lower aliphatic alcohols and water, contains 15-20% water) were purchased from Sigma-Aldrich.

Characterization. Transmission electron microscopy (TEM), high-angle annular dark-field scanning transmission electron microscopy (HAADF-STEM) and STEM energy-dispersive X-ray spectroscopy (HAADF-STEM-EDS) were collected on an FEI Talos F200X microscope at an accelerating voltage of 200 kV. The aberration-corrected HADDF-STEM and corresponding EDS analysis were carried out on an FEI Themis Z with a probe aberration corrector at the Feringa Nobel Prize Scientist Joint Research Center. Power X-ray diffraction (XRD) patterns were obtained using a D8 Advance X-ray Powder Diffractometer (Bruker) with Cu-Kα radiation (λ = 0.15406 nm). The Pt loading and atomic ratio of Pt_xNi_y/C were determined by the inductively coupled plasma atomic emission spectrometer (ICP-AES, Agilent 725). X-ray photoelectron spectroscopy (XPS) was performed on a Thermo Scientific ESCALAB 250Xi X-ray photoelectron spectrometer employing a monochromated Al-Kα X-ray source. The Raman spectrum was recorded on an inVia Reflex (Renishaw) with laser excitation at 532 nm.

The C K-edge XANES was obtained at beamline BL-17C of the National Synchrotron Radiation Research Center (NSRRC, Taipei). The Pt L_3-edge XAFS of com-Pt/C, Pt_1Ni_1/C, Pt_3Ni/C, and standard references were performed at the BL14W1 beamline of Shanghai Synchrotron Radiation Facility (SSRF, Shanghai) at ambient condition. The raw XAFS data were processed using the

Demeter package, and all spectra were normalized to achieve a unit edge jump.

After the stability tests, the catalysts were collected and removed from the electrode with the assistance of ultrasonication in ethanol for further characterization.

AIMD calculation details. To investigate the anchoring effect of Pt nanoparticles on graphene with and without defects, AIMD simulations were conducted using VASP with the RPBE[1,2] exchange correlation (XC) functional, along with the D3 dispersion correction scheme[3]. Our simulations employed a plane-wave cutoff of 400 eV and a Gaussian smearing of width 0.1 eV. The electronic structure was relaxed until all forces converged to less than 0.05 eV/Å. We conducted Γ-point AIMD simulations with a 1 fs time step and used a Nosé thermostat set at 300 K. As shown in Fig. 5 the presence of defects in graphene enables the formation of chemical bonds between Pt nanoparticles and graphene, thereby giving the anchoring effect of the carbon support on Pt nanoparticles.

Supplementary Notes

Pt-to-Ni ratio after electrochemical dealloying. We first construct a sphere model with radius (r) of ~1.5 nm, based on the average size of Pt_1Ni_1/C nanoparticles. The volume of a single particle ($V_{particle}$) is calculated as follows:

$$V_{\text{particle}} = \frac{4}{3}\pi r^3 = \frac{4}{3}\pi(1.5)^3 \approx 14.1\ \text{nm}^3$$

Then, the average atom volume (V_{atom}) can be estimated from unit cell volume, given that the volume of a face-centered cubic (fcc) unit cell is equal to the volume of four atoms:

$$V_{\text{atom}} = \frac{1}{4}{a_{\text{avg}}}^3$$

Where a_{avg} is the average lattice constant calculated from lattice constants of Pt (a_{Pt}) and Ni (a_{Ni}):

$$a_{\text{avg}} = \frac{1}{2}(a_{\text{Pt}} + a_{\text{Ni}}) = \frac{1}{2}(0.392 + 0.352) = 0.372\ \text{nm}$$

Therefore, the average atom volume (V_{atom}) is:

$$V_{\text{atom}} = \frac{1}{4}(0.372)^3 \approx 0.0129\ \text{nm}^3$$

The total number of atoms (N_{total}) in a single particle can be estimated as:

$$N_{\text{total}} = \frac{V_{\text{particle}}}{V_{\text{atom}}} \approx \frac{14.1}{0.0129} \approx 1093$$

Because the Pt-to-Ni ratio is ~1:1 before dealloying, thus, there are ~547 Pt atoms (N_{Pt}=547) and ~547 Ni atoms (N_{Ni}=547) in a single particle.

Assuming all the Ni atoms in the outermost monolayer dissolved after electrochemical dealloying while all the Pt atoms remain. Given that the thickness of the outermost monolayer can be estimated as one atomic layer, which is a_{avg}, and the volume of the outermost monolayer (V_{out}) can be calculated as follows:

$$V_{\mathrm{out}} = \frac{4}{3}\pi\left((r)^3 - \left(r - a_{\mathrm{avg}}\right)^3\right) \approx \frac{4}{3}\pi((1.5)^3 - (1.5 - 0.372)^3) \approx 8.13\ \mathrm{nm}^3$$

The dissolved number of Ni atoms ($N_{diss\text{-}Ni}$) in the outermost monolayer is:

$$N_{\mathrm{diss-Ni}} = \frac{1}{2} \times \frac{V_{\mathrm{out}}}{V_{\mathrm{avg}}} \approx \frac{1}{2} \times \frac{8.13}{0.0129} \approx 315$$

The number of Ni ($N_{core\text{-}Ni}$) that remain in the Pt_1Ni_1 core is:

$$N_{\mathrm{core-Ni}} = N_{\mathrm{Ni}} - N_{\mathrm{diss-Ni}} \approx 547 - 315 \approx 232$$

The Pt-to-Ni ratio ($\mathrm{ratio}_{Pt\text{-}to\text{-}Ni}$) across the dealloyed Pt_1Ni_1@Pt nanoparticle can be estimated as:

$$\mathrm{ratio}_{\mathrm{Pt-to-Ni}} = N_{\mathrm{Pt}}: N_{\mathrm{core-Ni}} \approx 547: 232 \approx 70: 30$$

It can be seen that in the sphere model, the Pt-to-Ni ratio in the Pt_1Ni_1 core remains 50:50, but the estimated Pt-to-Ni ratio across the entire particle increases to ~70:30. Notably, we assumed that all the Ni atoms in the outermost monolayer dissolved while the Pt atoms did not dissolve during the electrochemical dealloying process, so the Pt-to-Ni ratio of 70:30 is an overestimate. Indeed, the Pt-to-Ni ratio (62.3:37.7) obtained from EDS analysis of dealloyed Pt_1Ni_1@Pt/C appears to be more reasonable. This analysis suggests that the Pt-to-Ni ratio in the Pt_1Ni_1 core remains 50:50, in line with theoretical predictions.

Supplementary Figures

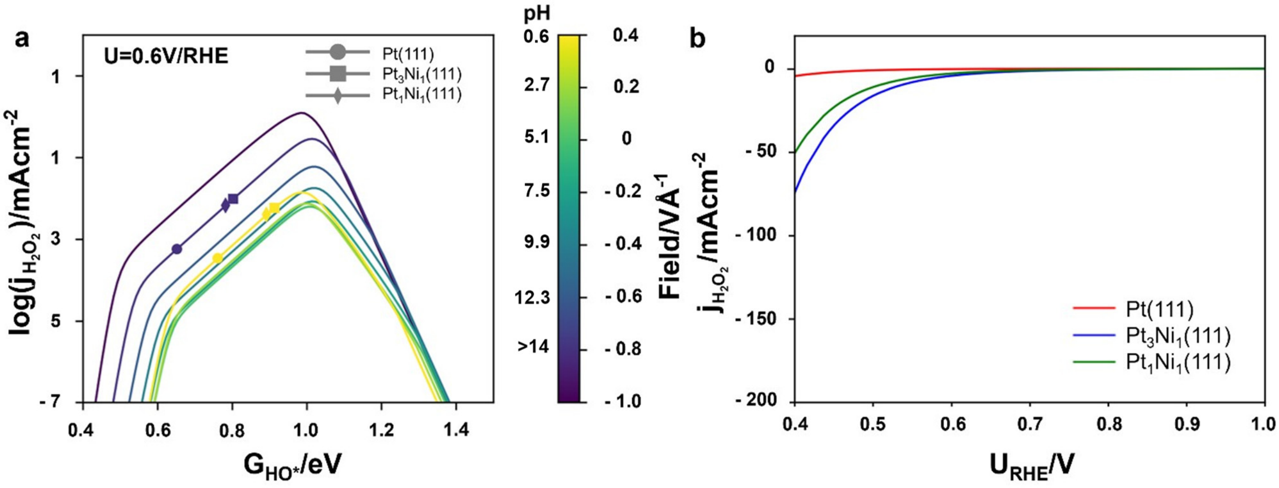

Supplementary Fig. 1 | Simulated ORR activities of Pt(111) and Pt_xNi_y@Pt(111) based on pH-dependent microkinetic modeling. a pH-dependent $2e^-$ ORR volcano activity model as a function of HO* binding free energy, where a lower electric field corresponds to a lower pH environment. **b** $2e^-$ ORR polarization curves simulated at pH≈0.

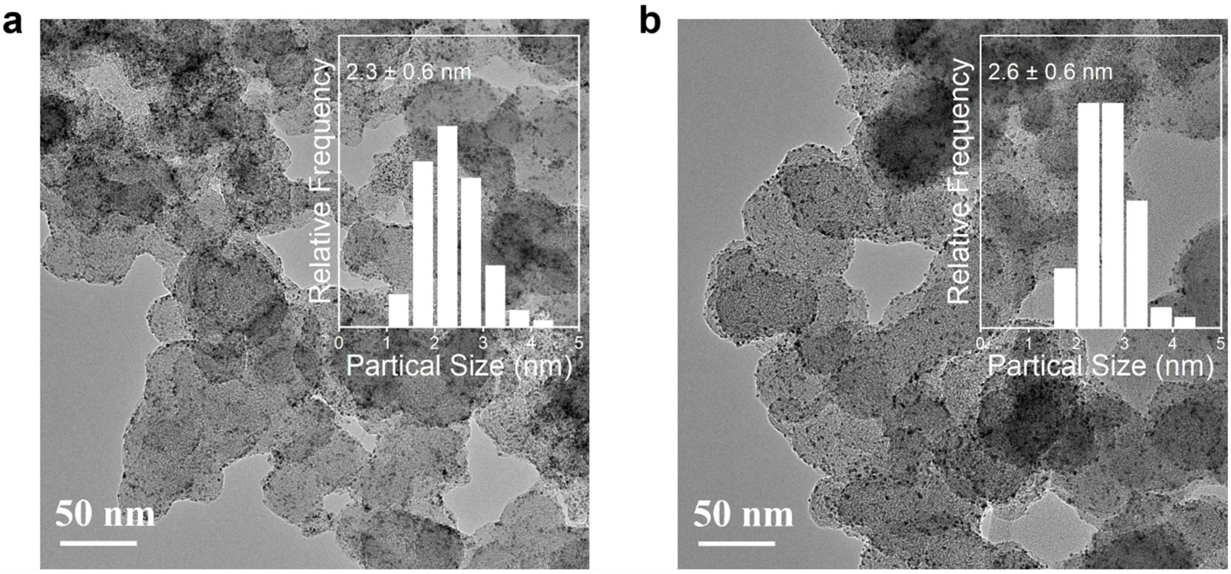

Supplementary Fig. 2 | Particle size distribution of synthesized NPs. TEM images and corresponding size distribution of **a** syn-Pt/C and **b** Pt_3Ni_1/C.

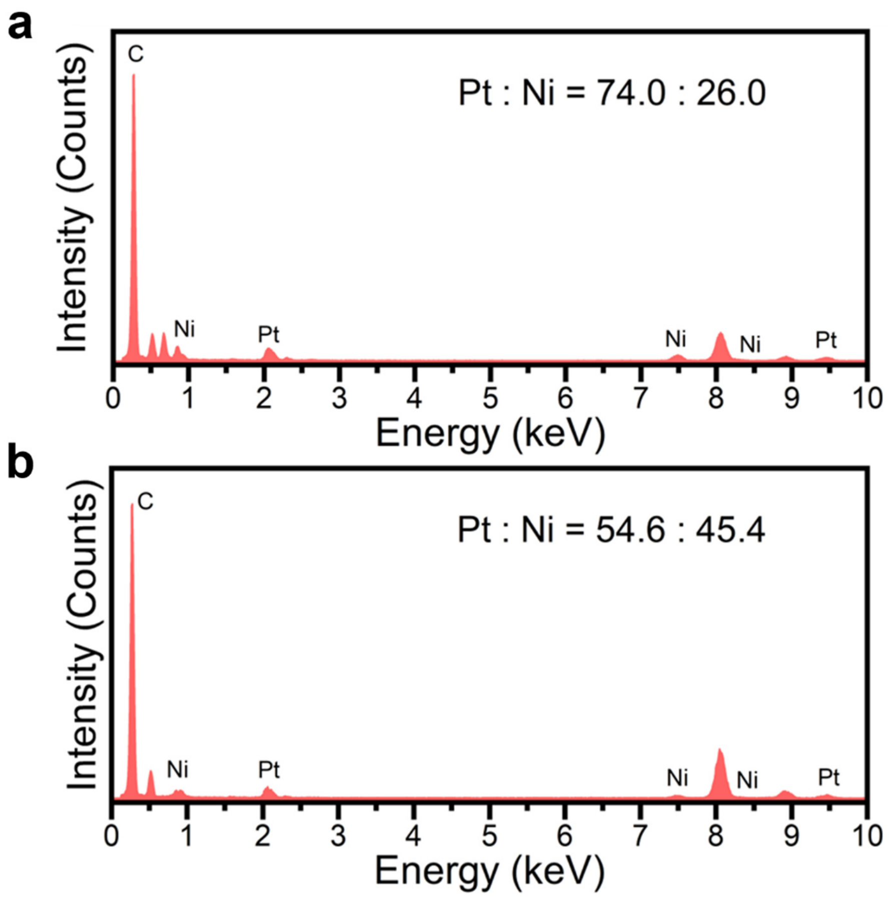

Supplementary Fig. 3 | Elemental analysis of different Pt_xNi_y/C NPs by EDS. EDS spectrum of **a** Pt_3Ni_1/C and **b** Pt_1Ni_1/C.

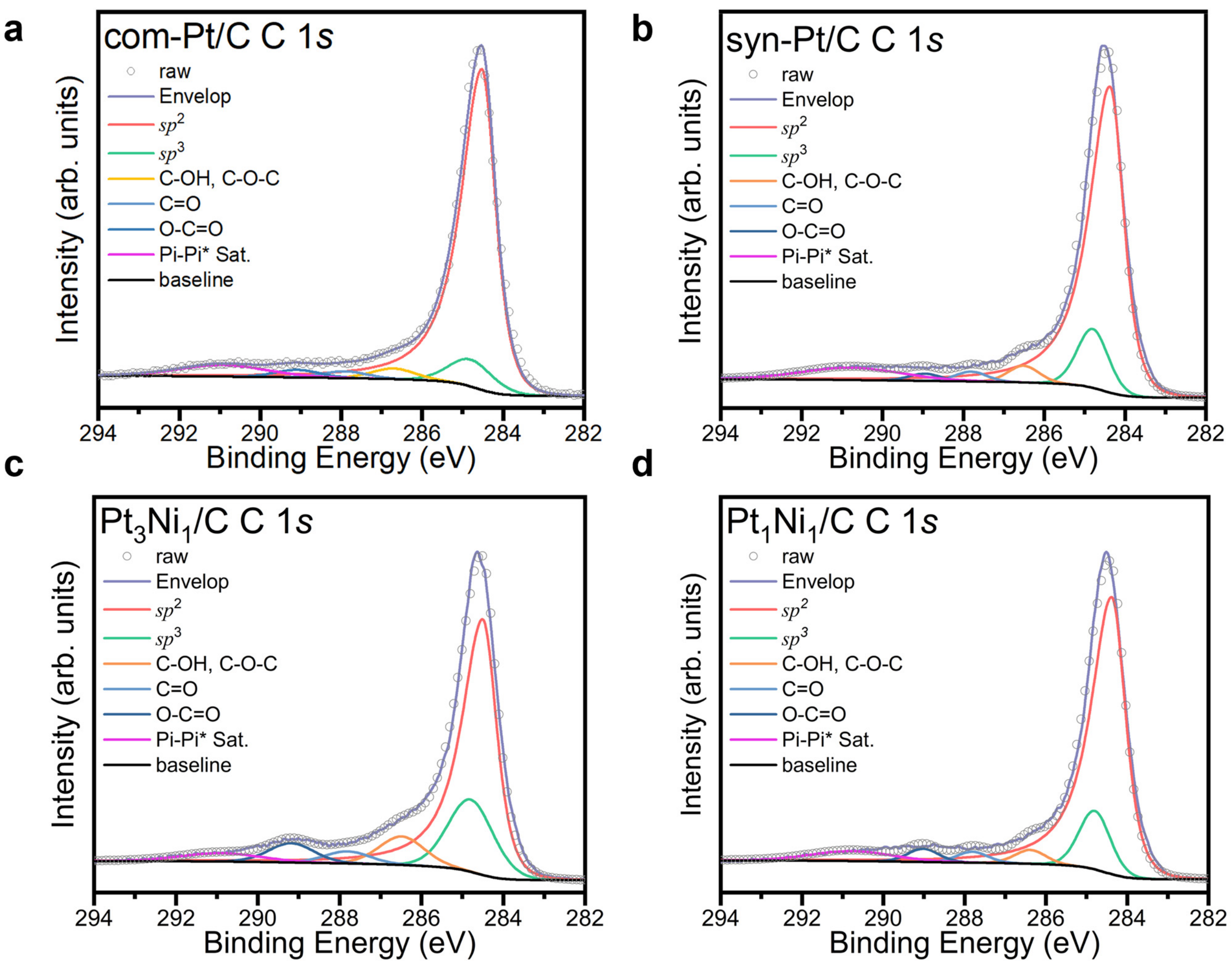

Supplementary Fig. 4 | Validation of defective carbon substrates. XPS C1s spectrum of **a** com-Pt/C, **b** syn-Pt/C, **c** Pt_3Ni_1/C, and **d** Pt_1Ni_1/C.

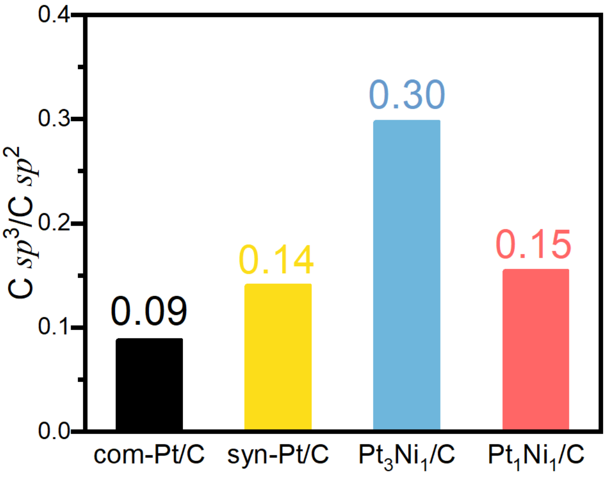

Supplementary Fig. 5 | Comparison of carbon defect density among different samples. *sp*^{3}-/*sp*^{2}-hybridized carbon ratio of com-Pt/C, syn-Pt/C, Pt_3Ni_1/C, and Pt_1Ni_1/C calculated from C 1s XPS spectra.

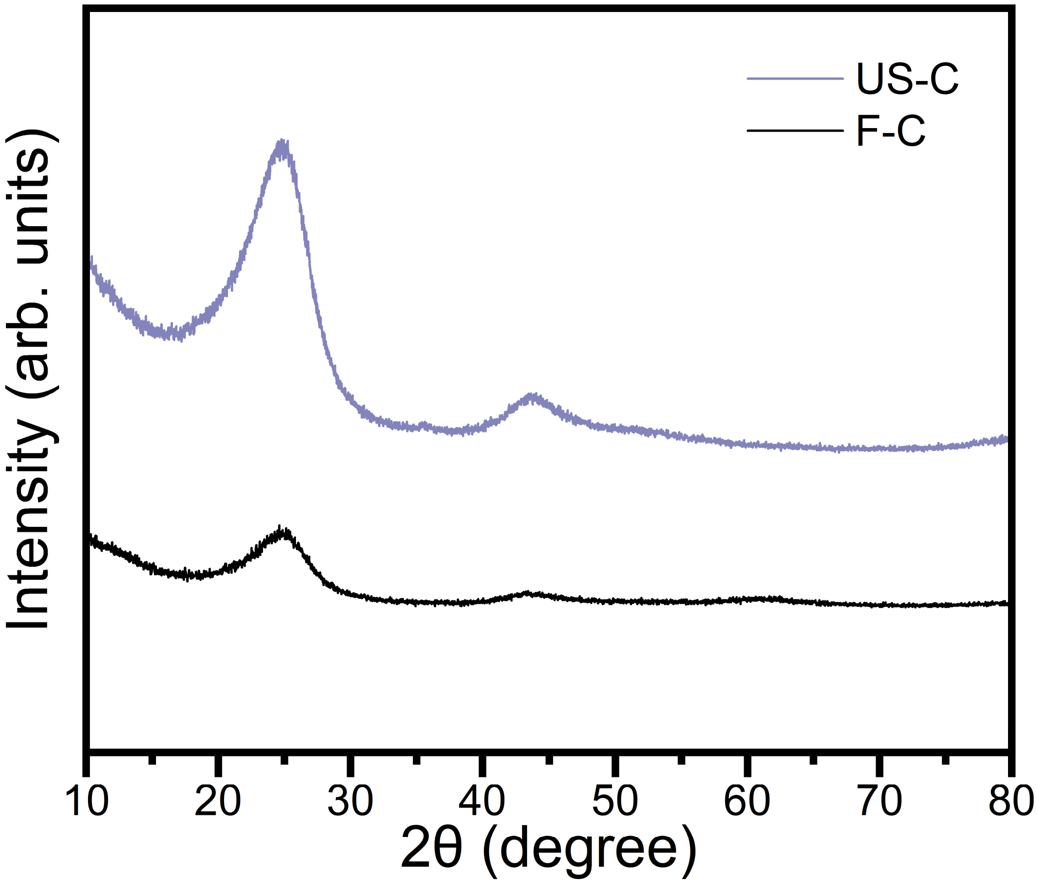

Supplementary Fig. 6 | Phase transition of carbon substrate. XRD spectra of F-C and US-C.

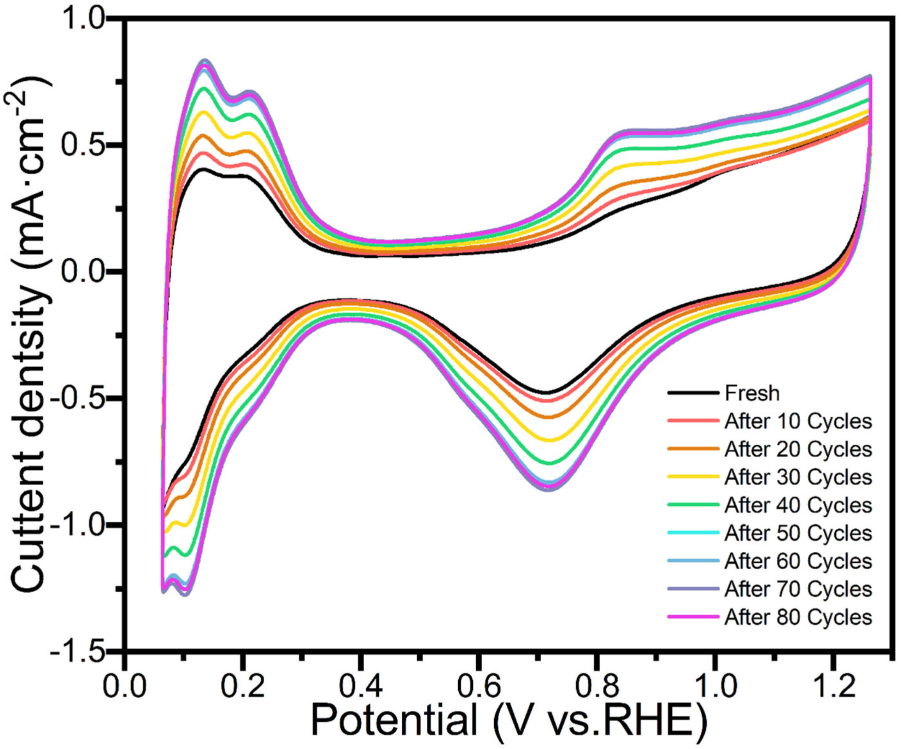

Supplementary Fig. 7 | Formation of core-shell structures. CV curves of Pt_1Ni_1/C NPs with different electrochemical dealloying cycles in 0.1 M $HClO_4$ solution.

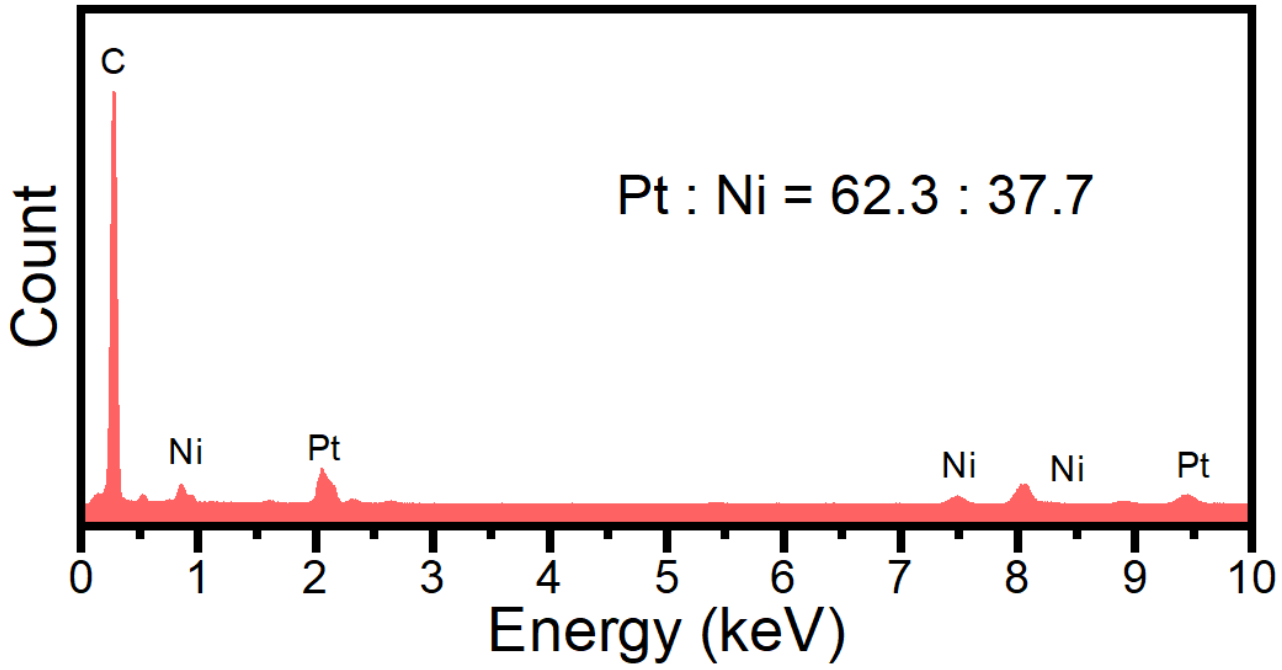

Supplementary Fig. 8 | Composition after electrochemical dealloying. EDS spectrum of Pt_1Ni_1@Pt/C.

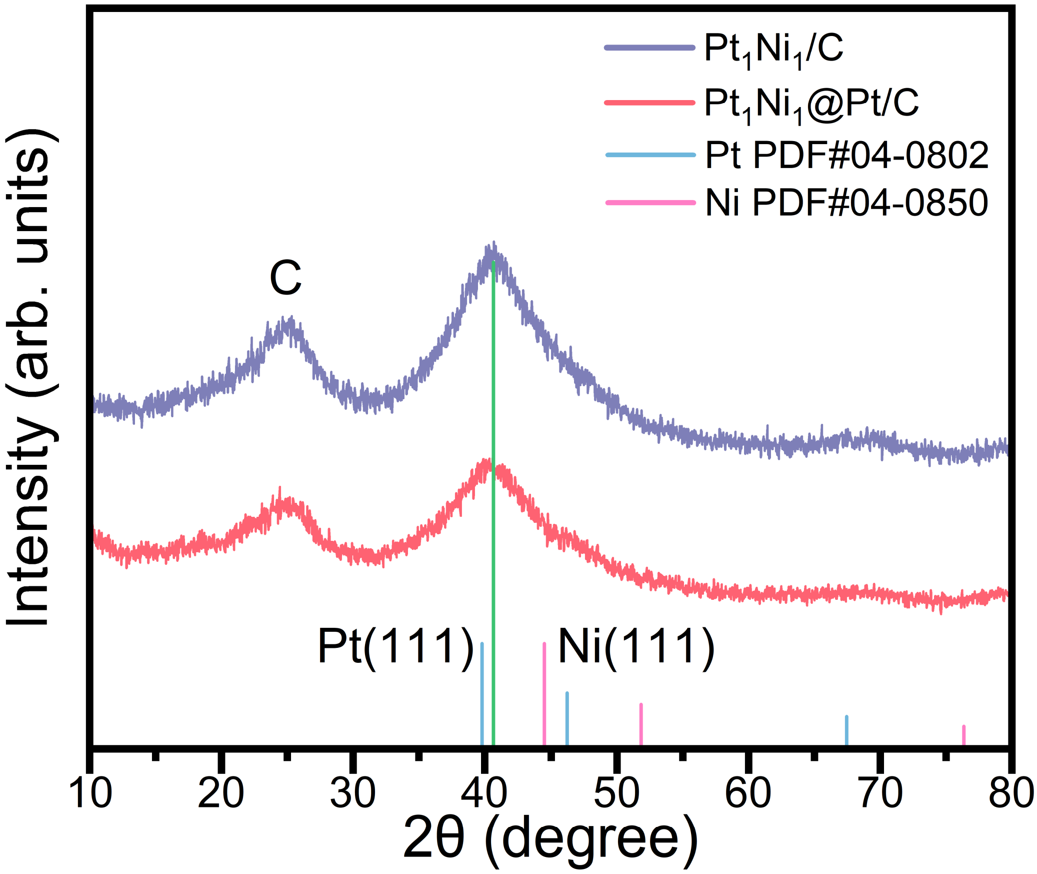

Supplementary Fig. 9 | Structure characterizations. XRD patterns of Pt_1Ni_1/C and $Pt_1Ni_1@Pt/C$. The peak around 2θ ≈ 25° is assigned to carbon matrix the same as that in Supplementary Fig. 6.

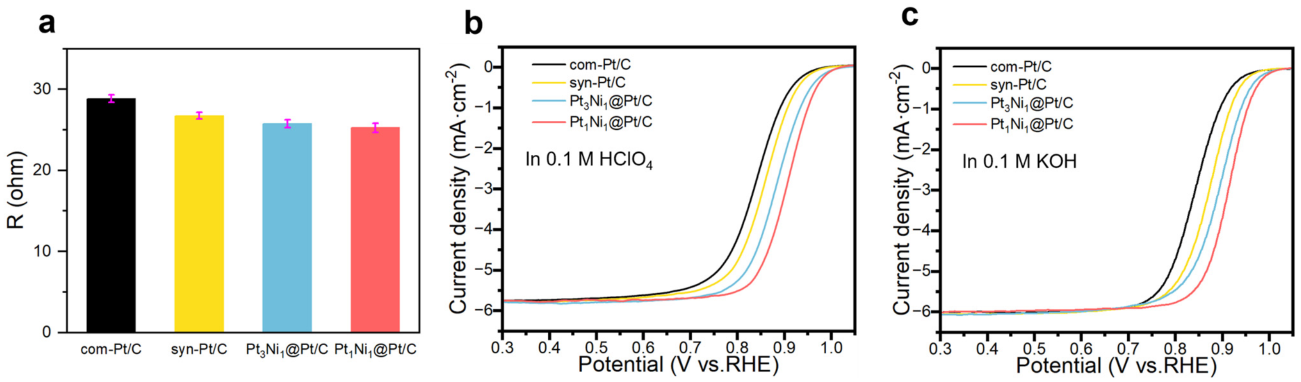

Supplementary Fig. 10 | ORR performance. **a** Solution resistance obtained by EIS. Non-*i*R corrected ORR polarization curves of different catalysts measured in **b** 0.1 M $HClO_4$ and **c** 0.1 M KOH. Electrode area: 0.196 cm^2.

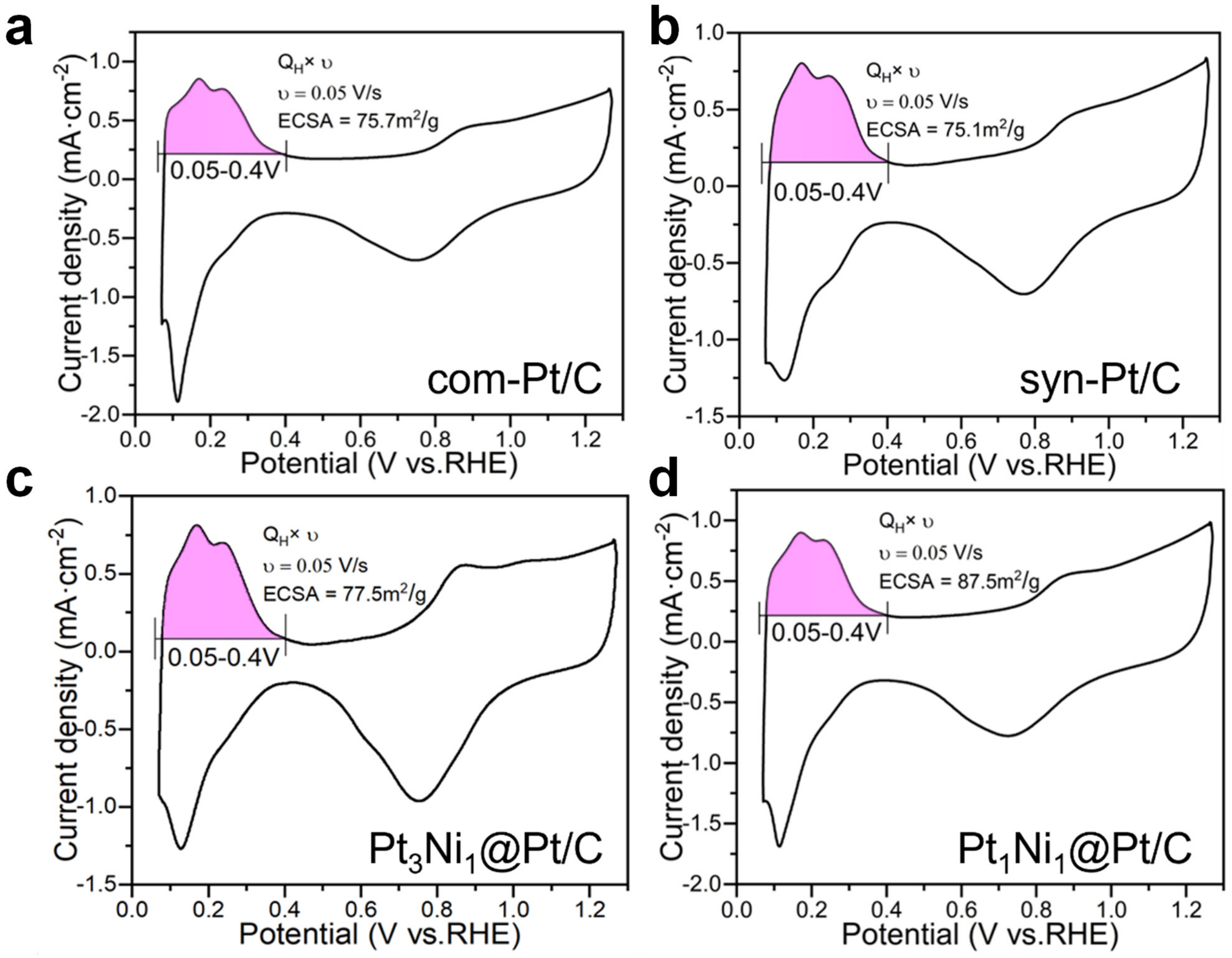

Supplementary Fig. 11 | ECSA measurements and calculations. CV curves and corresponding calculated ECSA of **a** com-Pt/C **b** syn-Pt/C **c** Pt_3Ni_1@Pt/C and **d** Pt_1Ni_1@Pt/C based on the hydrogen underpotential deposition (HUPD).

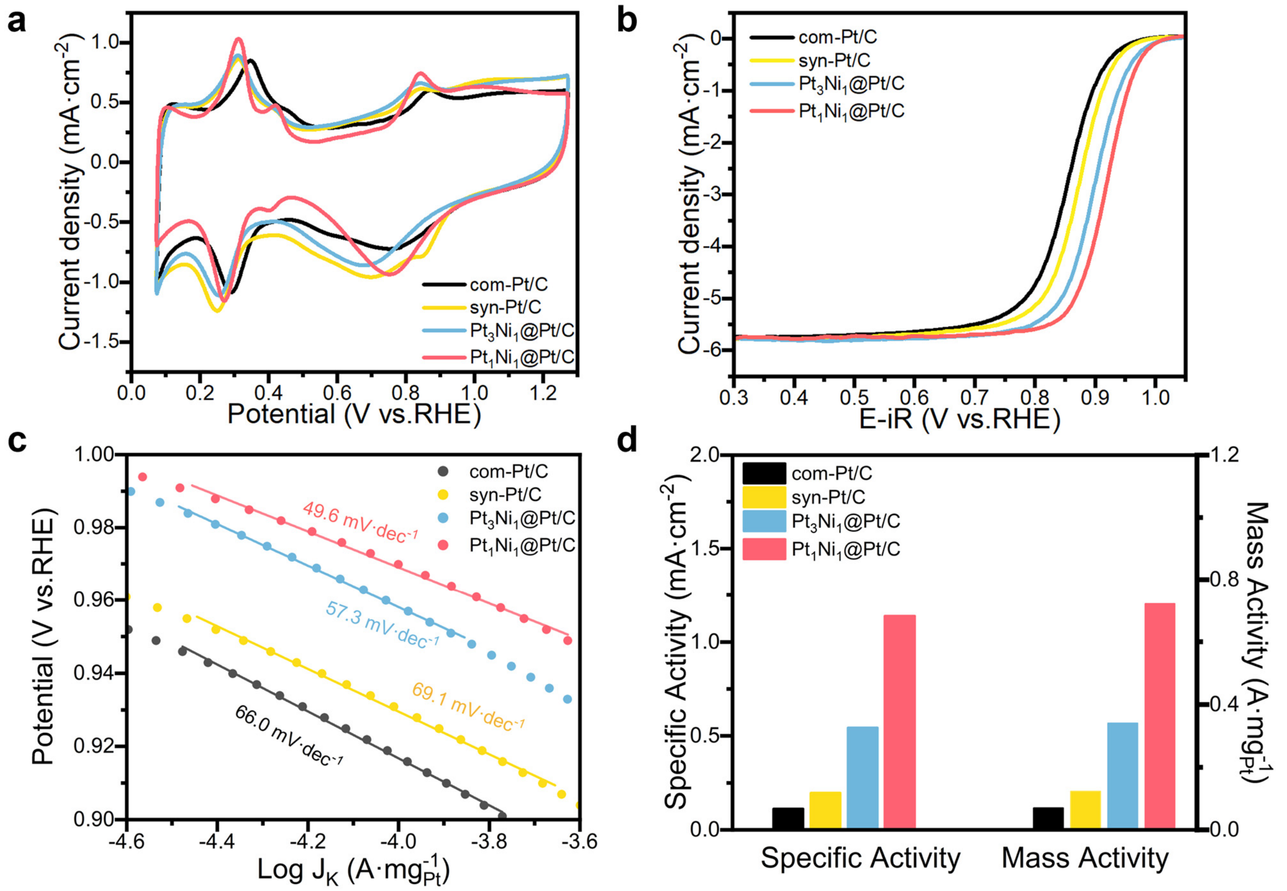

Supplementary Fig. 12 | ORR performance in alkaline media. **a** CV curves **b** *i*R corrected ORR polarization curves **c** Tafel plots and **d** specific and mass activities of different catalysts measured in 0.1 M KOH. Electrode area: 0.196 cm^2. *i*R correction was conducted at 100% manually.

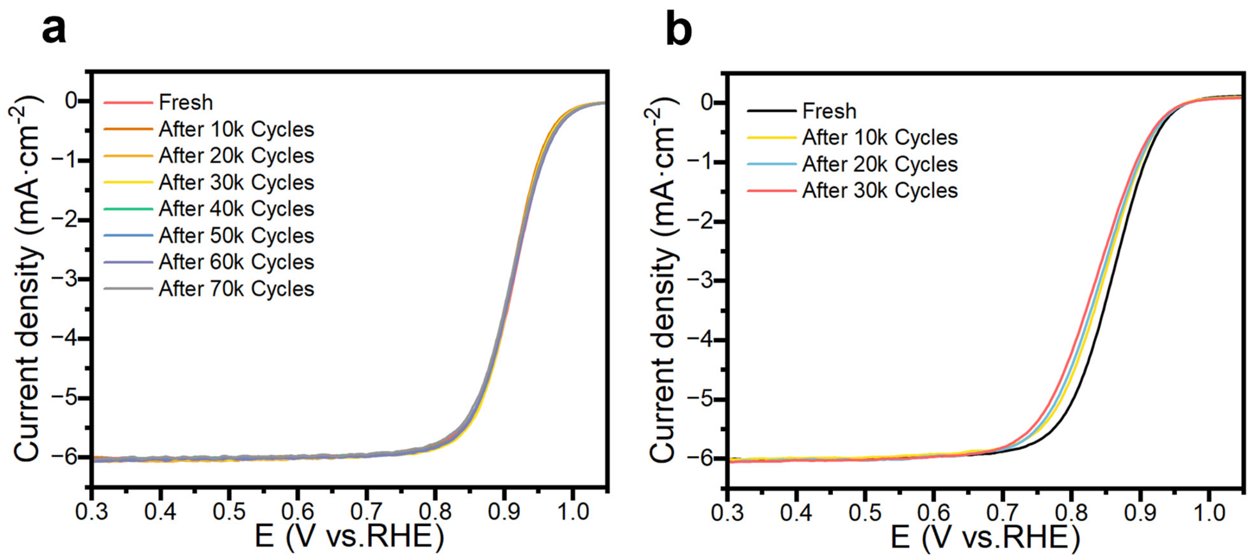

Supplementary Fig. 13 | ORR durability of Pt_1Ni_1@Pt/C and com-Pt/C. a Non-*i*R corrected ORR polarization curves of Pt_1Ni_1@Pt/C after every 10k potential cycles. **b** Non-*i*R corrected ORR polarization curves of com-Pt/C after every 10k potential cycles.

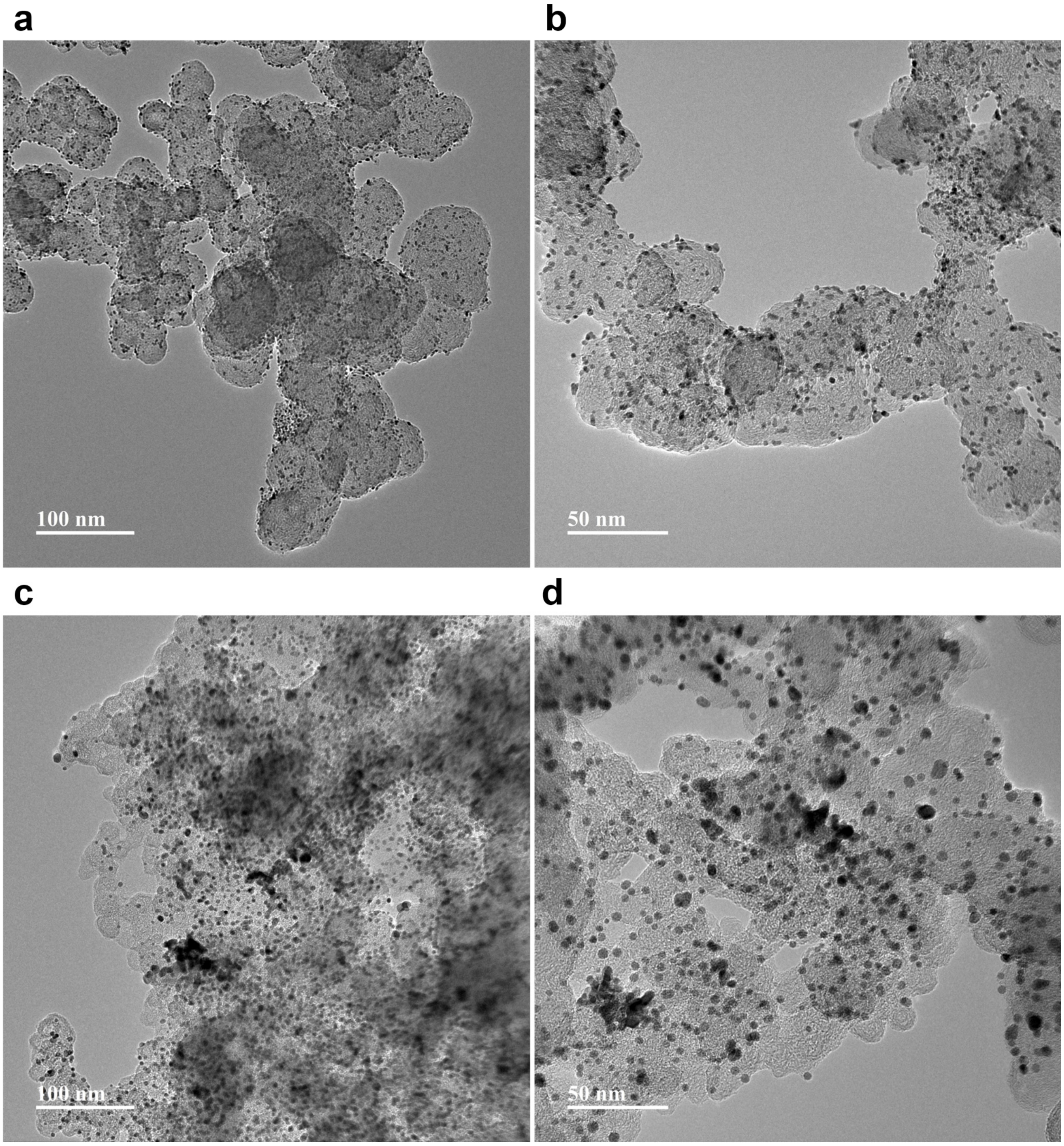

Supplementary Fig. 14 | Structure stability of com-Pt/C. TEM images of **a**, **b** fresh com-Pt/C and **c**, **d** com-Pt/C after 30k ADT cycles.

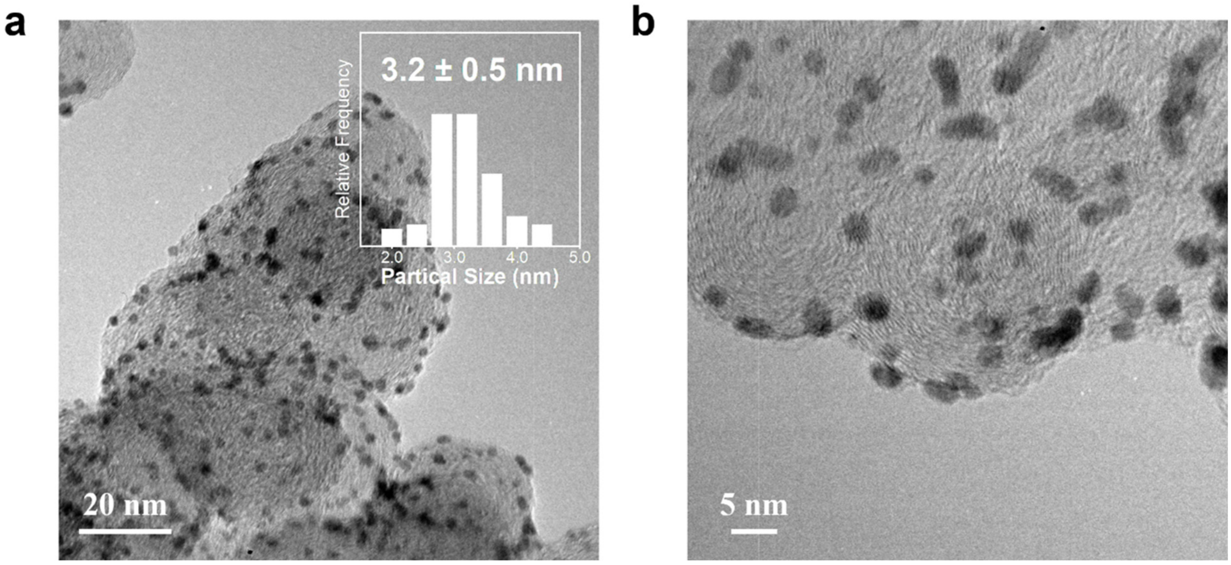

Supplementary Fig. 15 | Structure stability of Pt_1Ni_1@Pt/C NPs. a TEM and **b** HRTEM images of Pt_1Ni_1@Pt/C NPs after 70k ADT cycles.

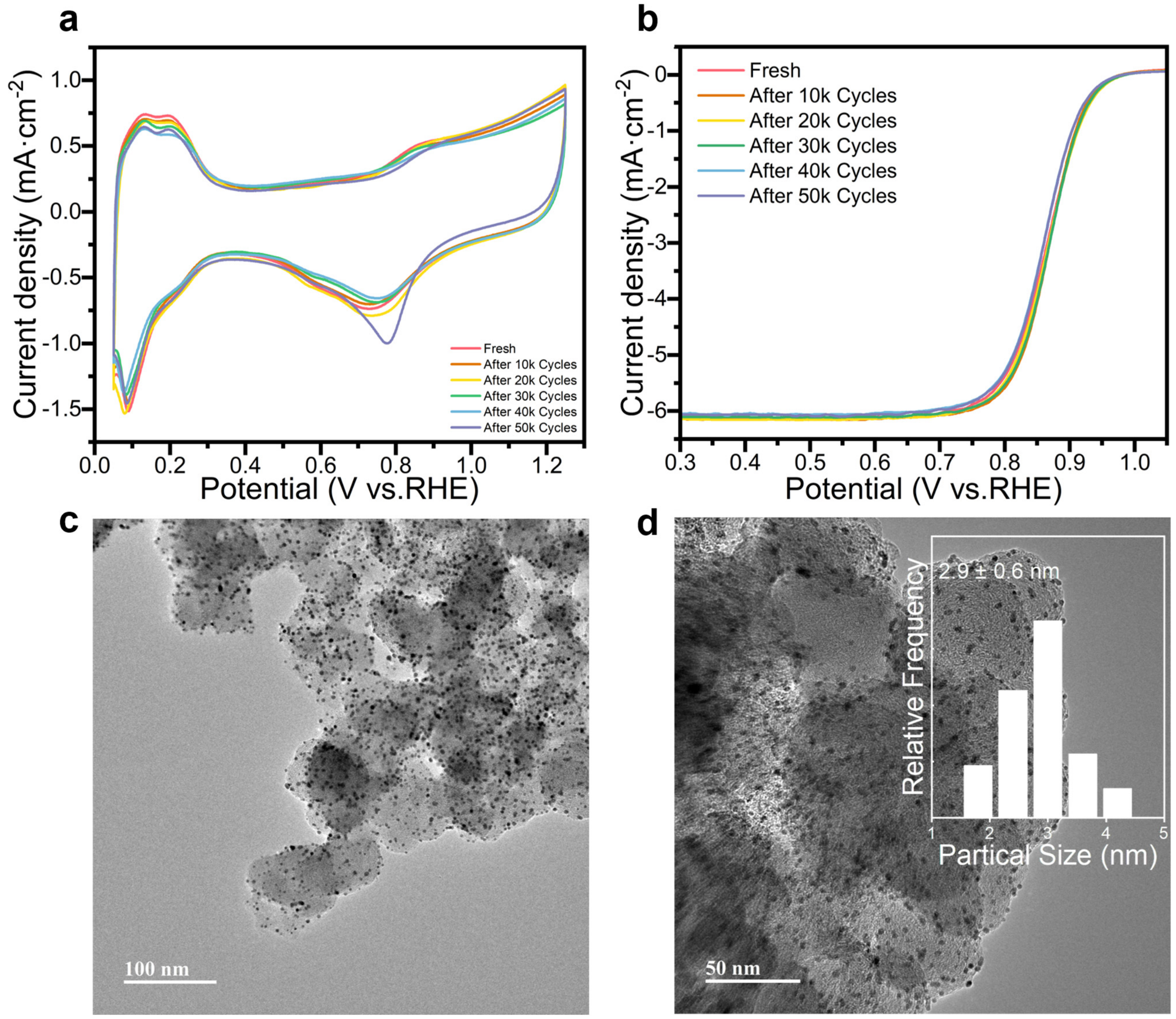

Supplementary Fig. 16 | Durability test of syn-Pt/C. a CV curves **b** ORR polarization curves of syn-Pt/C after different ADT cycles. **c** and **d** TEM images of syn-Pt/C after 50k ADT cycles. Negligible degradation is observed during ADT test. The aggregation of NPs is significantly inhibited, and the average size slightly increases to 2.9 nm.

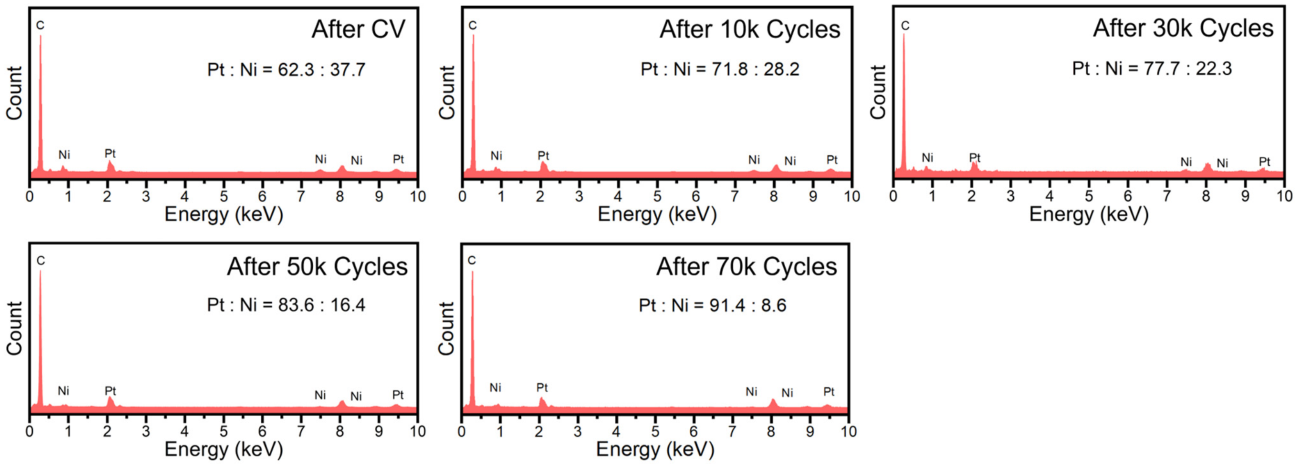

Supplementary Fig. 17 | Composition changes during ADT test. EDS spectra of Pt_1Ni_1@Pt/C after CV (electrochemical dealloying) and different ADT cycles.

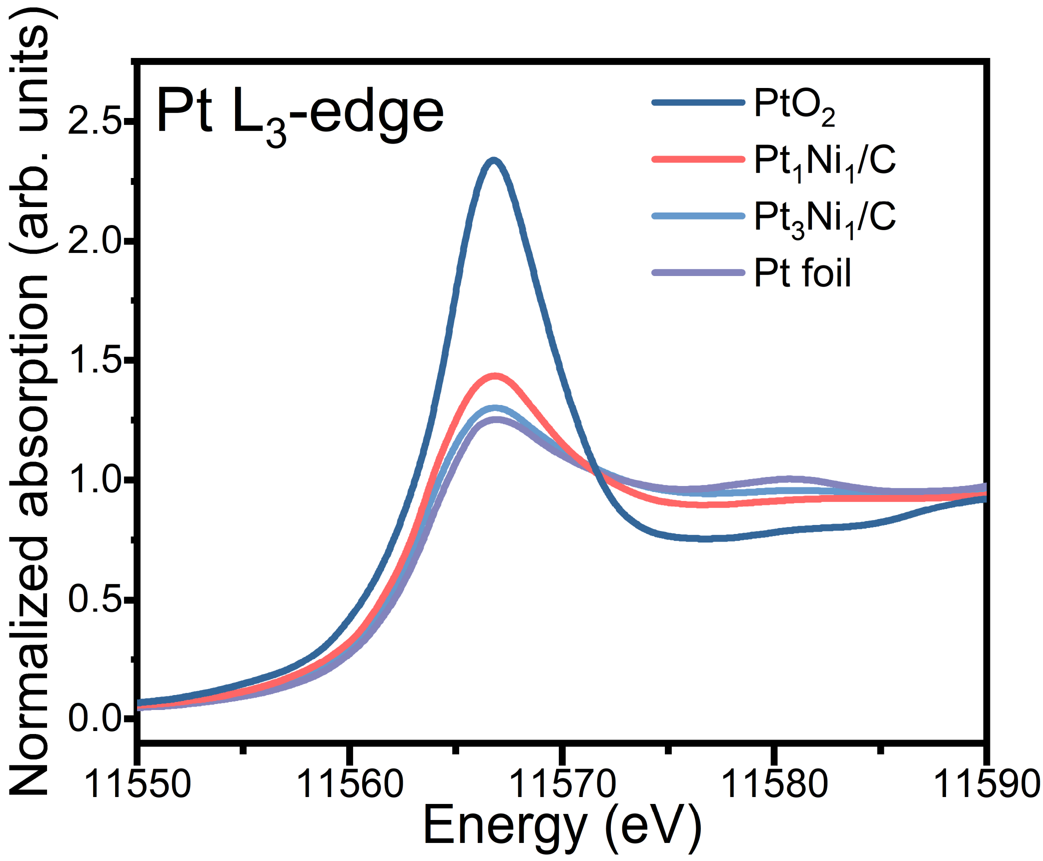

Supplementary Fig. 18 | Validation of Pt-C interaction. XANES spectra at the Pt L_3-edge of Pt_1Ni_1/C, Pt_3Ni_1/C, standard Pt foil, and standard PtO_2.

Supplementary Tables

Supplementary Table 1 | Composition of Pt_xNi_y/C NPs calculated from ICP-OES results.

Samples	Pt : Ni (Molar ratio)	Weight %	
		Pt	Ni
Pt/C	/	10.92	/
Pt_3Ni_1/C	2.84	11.56	1.23
Pt_1Ni_1/C	0.99	14.6	4.4

Supplementary Table 2 | ORR electrocatalytic performance of different catalysts measured in 0.1 M $HClO_4$.

Catalysts	Tafel slope (mV/dec)	Half-wave potential (V)	ECSA (m^2/g_{Pt})	Specific activity (mA/cm_{Pt}^2)	Mass activity (A/mg_{Pt})
com-Pt/C	72.2±4.5	0.858±0.005	74.8±0.9	0.172±0.022	0.157±0.042
syn-Pt/C	55.8±3.1	0.872±0.005	73.9±3.1	0.222±0.004	0.194±0.061
Pt_3Ni_1@Pt/C	52.0±1.6	0.908±0.002	79.0±1.4	0.727±0.092	0.559±0.038
Pt_1Ni_1@Pt/C	44.9±1.1	0.927±0.001	87.2±0.3	1.554±0.027	1.424±0.019

*Specific activity and mass activity are calculated at 0.9 V vs. RHE.

*All data was collected by at least 3 independent measurements.

Supplementary Table 3 | ORR electrocatalytic performance of different catalysts measured in 0.1 M KOH.

Catalysts	Tafel slope (mV/dec)	Half-wave potential (V)	ECSA (m^2/g_{Pt})	Specific activity (mA/cm_{Pt}^2)	Mass activity (A/mg_{Pt})
com-Pt/C	66.0	0.842	62.1	0.113	0.070
syn-Pt/C	69.1	0.798	62.6	0.197	0.123
Pt_3Ni_1@Pt/C	57.3	0.886	62.6	0.544	0.341
Pt_1Ni_1@Pt/C	49.5	0.914	63.6	1.137	0.723

* Specific activity and mass activity are calculated at 0.9 V vs. RHE.

Supplementary Table 4 | Durability comparison with recently reported catalysts measured in an RDE set-up.

Catalyst	Rotation speed (rpm)	Temp. (°C)	Pt loading (μg/cm^2)	Electrolyte	Potential range (V *vs.* RHE) @scan rate (mV/s)	ADT cycles	MA retention %	Reference
Pt_1Ni_1@Pt/C	1600	25	15	O_2- saturated 0.1 M $HClO_4$	0.6-1.0@100	70000	98.4	This work
PtCo@NGNS	1600	25	25	O_2- saturated 0.1 M $HClO_4$	0.6-1.0@100	30000	80	4
Pt/Fe–N–C	1600	25	15.3	O_2- saturated 0.05 M H_2SO_4	0.6-1.0@50	10000	81	5
fct-PtFeIr/C	1600	25	10	O_2- saturated 0.1 M $HClO_4$	0.6-1.1@100	10000	88.9	6
PtNi-BNCs/C	1600	25	6.8	O_2- saturated 0.1 M $HClO_4$	0.6-1.1@50	50000	86.3	7
$Pt_{4.31}$Ga NWs/C	1600	25	12.75	O_2- saturated 0.1 M $HClO_4$	0.6-1.1@100	30000	84.2	8
Pt@Pt-skin Pt_3Ni CSFW/C	1600	RT	6.5	O_2- saturated 0.1 M $HClO_4$	0.6-1.0@100	50000	96.9	9
Ni_3C/Pt_3Ni	1600	25	18.64	O_2- saturated 0.1 M $HClO_4$	0.6-1.0@100	10000	93	10
fct-Pt-Co@Pt/C	1600	25	10.2	O_2- saturated 0.1 M $HClO_4$	0.6-1.1@100	30000	79	11
$Pt_{1.3}$Ni NWs,	1600	25	27.6	O_2- saturated 0.1 M $HClO_4$	0.6-1.0@100	10000	90.5	12
$L1_0$-FePt	1600	25	40	O_2- saturated 0.1 M $HClO_4$	0.6-1.0@100	30000	91	13
PtPb nanoparticles/C	1600	25	6.37	O_2- saturated 0.1 M $HClO_4$	0.6-1.1@100	50000	92.7	14
Mo-Pt_3Ni/C	1600	25	4.08	O_2- saturated 0.1 M $HClO_4$	0.6-1.1@50	8000	90.3	15
O-$PtCo_3$@HNCS	1600	25	20.2	O_2- saturated	0.6-1.0@100	20000	92.6	16

				0.1 M $HClO_4$				
PtNiN	1600	25	20	O_2- saturated 0.1 M $HClO_4$	0.6-1.0@50	30000	81	17
Pt-skin Pt_3Fe z-NWs	1600	25	5	O_2- saturated 0.1 M $HClO_4$	0.6-1.1@50	50000	47.8	18
$Pd_{1.8}Pt$ tesseracts	1600	25	20	O_2- saturated 0.1 M $HClO_4$	0.6-1.1@100	10000	92.6	19
$PtPb_{1.07}Ni_{0.10}$ octahedra/C	1600	RT	12.85	O_2- saturated 0.1 M $HClO_4$	0.6-1.0@50	15000	79.1	20
fct-PtFe/C	1600	25	7.8	O_2- saturated 0.1 M $HClO_4$	0.6-1.0@50	10000	96.6	21
PtNiRh NWs/C	1600	25	15	O_2- saturated 0.1 M $HClO_4$	0.6-1.0@50	10000	87.2	22

*Temp. represents temperature.

*RT represents room temperature.

Supplementary References

1. Hammer, B., Hansen, L. B. & Nørskov, J. K. Improved adsorption energetics within density-functional theory using revised Perdew-Burke-Ernzerhof functionals. *Phys. Rev. B* **59**, 7413-7421, (1999).
2. Perdew, J. P., Burke, K. & Ernzerhof, M. Generalized gradient approximation made simple. *Phys. Rev. Lett.* **77**, 3865-3868, (1996).
3. Grimme, S., Antony, J., Ehrlich, S. & Krieg, H. A consistent and accurate ab initio parametrization of density functional dispersion correction (DFT-D) for the 94 elements H-Pu. *J. Chem. Phys.* **132**, 154104, (2010).
4. Zaman, S. *et al.* Scalable Molten Salt Synthesis of Platinum Alloys Planted in Metal-Nitrogen-Graphene for Efficient Oxygen Reduction. *Angew. Chem. Int. Ed.* **61**, e202115835, (2022).
5. Xiao, F. *et al.* Fe–N–C Boosts the Stability of Supported Platinum Nanoparticles for Fuel Cells. *J. Am. Chem. Soc.* **144**, 20372-20384, (2022).
6. Yang, Z., Yang, H., Shang, L. & Zhang, T. Ordered PtFeIr Intermetallic Nanowires Prepared through a Silica-Protection Strategy for the Oxygen Reduction Reaction. *Angew. Chem. Int. Ed.* **61**, 202113278, (2022).
7. Tian, X. *et al.* Engineering bunched Pt-Ni alloy nanocages for efficient oxygen reduction in practical fuel cells. *Science* **366**, 850-856, (2019).
8. Gao, L. *et al.* Unconventional p-d Hybridization Interaction in PtGa Ultrathin Nanowires Boosts Oxygen Reduction Electrocatalysis. *J. Am. Chem. Soc.* **141**, 18083-18090, (2019).
9. Jin, H. *et al.* Mesoporous Pt@Pt-skin Pt_3Ni core-shell framework nanowire electrocatalyst for efficient oxygen reduction. *Nat. Commun.* **14**, 1518, (2023).
10. Ding, H. *et al.* Epitaxial Growth of Ultrathin Highly Crystalline Pt–Ni Nanostructure on a Metal Carbide Template for Efficient Oxygen Reduction Reaction. *Adv. Mater.* **34**, 2109188, (2022).
11. Xie, M. *et al.* Pt-Co@Pt Octahedral Nanocrystals: Enhancing Their Activity and Durability toward Oxygen Reduction with an Intermetallic Core and an Ultrathin Shell. *J. Am. Chem. Soc.* **143**, 8509-8518, (2021).
12. Ma, Y. *et al.* Platinum-Based Nanowires as Active Catalysts toward Oxygen Reduction Reaction: In Situ Observation of Surface-Diffusion-Assisted, Solid-State Oriented Attachment. *Adv. Mater.* **29**, 1703460, (2017).
13. Li, J. *et al.* Fe Stabilization by Intermetallic $L1_0$-FePt and Pt Catalysis Enhancement in $L1_0$-FePt/Pt Nanoparticles for Efficient Oxygen Reduction Reaction in Fuel Cells. *J. Am. Chem. Soc.* **140**, 2926-2932, (2018).
14. Bu, L. *et al.* Biaxially strained PtPb/Pt core/shell nanoplate boosts oxygen reduction catalysis. *Science* **354**, 1410-1414, (2016).
15. Huang, X. *et al.* High-performance transition metal–doped Pt_3Ni octahedra for oxygen reduction reaction. *Science* **348**, 1230-1234, (2015).
16. Hu, Y., Guo, X., Shen, T., Zhu, Y. & Wang, D. Hollow Porous Carbon-Confined Atomically Ordered $PtCo_3$ Intermetallics for an Efficient Oxygen Reduction Reaction. *ACS Catal.* **12**, 5380-5387, (2022).
17. Song, L. *et al.* One-Step Facile Synthesis of High-Activity Nitrogen-Doped PtNiN Oxygen Reduction Catalyst. *ACS Appl. Energy Mater.* **5**, 5245-5255, (2022).
18. Luo, M. *et al.* Stable High-Index Faceted Pt Skin on Zigzag-Like PtFe Nanowires Enhances Oxygen Reduction Catalysis. *Adv. Mater.* **30**, 1705515, (2018).
19. Chen, S. *et al.* Pd-Pt Tesseracts for the Oxygen Reduction Reaction. *J. Am. Chem. Soc.* **143**, 496-503, (2021).
20. Bu, L. *et al.* PtPb/PtNi Intermetallic Core/Atomic Layer Shell Octahedra for Efficient Oxygen Reduction Electrocatalysis. *J. Am. Chem. Soc.* **139**, 9576-9582, (2017).

21. Chung, D. Y. *et al.* Highly Durable and Active PtFe Nanocatalyst for Electrochemical Oxygen Reduction Reaction. *J. Am. Chem. Soc.* **137**, 15478-15485, (2015).
22. Li, K. *et al.* One-Nanometer-Thick PtNiRh Trimetallic Nanowires with Enhanced Oxygen Reduction Electrocatalysis in Acid Media: Integrating Multiple Advantages into One Catalyst. *J. Am. Chem. Soc.* **140**, 16159-16167, (2018).
